# Responsiveness of soil nitrogen fractions and bacterial communities to afforestation in the Loess Hilly Region (LHR) of China

**DOI:** 10.1038/srep28469

**Published:** 2016-06-23

**Authors:** Chengjie Ren, Pingsheng Sun, Di Kang, Fazhu Zhao, Yongzhong Feng, Guangxin Ren, Xinhui Han, Gaihe Yang

**Affiliations:** 1 College of Agronomy, Northwest A&F University, Yangling, 712100 Shaanxi, China; 2 The Research Center of Recycle Agricultural Engineering and Technology of Shaanxi Province, Yangling 712100 Shaanxi, China; 3 College of Forestry, Northwest A&F University, Yangling, 712100 Shaanxi, China; 4 College of Urban and Environmental Science, Northwest University, Xi’an, 712100 Shaanxi, China

## Abstract

In the present paper, we investigated the effects of afforestation on nitrogen fractions and microbial communities. A total of 24 soil samples were collected from farmland (FL) and three afforested lands, namely Robinia pseudoacacia L (RP), Caragana korshinskii Kom (CK), and abandoned land (AL), which have been arable for the past 40 years. Quantitative PCR and Illumina sequencing of 16S rRNA genes were used to analyze soil bacterial abundance, diversity, and composition. Additionally, soil nitrogen (N) stocks and fractions were estimated. The results showed that soil N stock, N fractions, and bacterial abundance and diversity increased following afforestation. *Proteobacteria*, *Acidobacteria*, and *Actinobacteria* were the dominant phyla of soil bacterial compositions. Overall, soil bacterial compositions generally changed from *Actinobacteria* (*Acidobacteria*)-dominant to *Proteobacteria*-dominant following afforestation. Soil N fractions, especially for dissolved organic nitrogen (DON), were significantly correlated with most bacterial groups and bacterial diversity, while potential competitive interactions between *Proteobacteria* (order *Rhizobiales*) and *Cyanobacteria* were suggested. In contrast, nitrate nitrogen (NO_3_^−^-N) influenced soil bacterial compositions less than other N fractions. Therefore, the present study demonstrated that bacterial diversity and specific species respond to farmland-to-forest conversion and hence have the potential to affect N dynamic processes in the Loess Plateau.

With rising concern regarding climatic consequences, afforestation has often been proposed as an effective and cost-efficient mitigation response to climate change^1^,^2^. This widespread shift in ecosystem have strong potential to alter key ecosystem processes, the resultant alterations can affect N cycles at the ecosystem and regional scale^3^,^4^,^5^, further influence the forest N stock under the process of ecological restoration^6^. In fact, it has been estimated that soil nitrogen (N) accounts for approximately 88% of the global plant N demand^7^, and changes in soil N availability greatly affect plant growth and productivity, particularly for rooting and canopy closure^8^. Meanwhile, afforestation has been demonstrated to enhance soil N accumulation^9^,^10^ and alter the soil N availability for soil bacteria growth^11^,^12^, thereby potentially influencing the element cycles of terrestrial ecosystems^5^,^6^. Therefore, elucidating the response of soil N stocks and fractions to changes in the ecosystem, especially for below-ground ecosystems such as soil bacterial community, are essential to revealing the biogeochemical cycling process.

Soil N respond to the quantity and quality of soil organic matter (SOM) input with changes in the microbial process^13^,^14^. Changes in microbial communities are hypothesized to alter ecosystem functions, including N sequestration, N cycling, plant litter decomposition and soil N availability^3^,^6^,^15^. A variety of mechanism between the soil N and soil bacterial communities have been identified, including direct effects and indirect effects^16^. For example, soil bacteria decompose the SOM inputs, thereby changing the amount of dissolved organic nitrogen^12^. As such, soil nitrogen-fixing bacteria fix atmospheric N and accelerate the soil nitrogen transformation^16^,^17^. However, soil N could offer a greater amount of resources, such as NH_4_^+^ and NO_3_^−^, for soil bacteria to live on^18^ and also alter the below-ground carbon (C) allocation, hence influencing the amount of C available to the microbe. It is also shown that DON is the most active component of soil organic matter and responds more rapidly than soil organic fractions to afforestation, which lead to significant modifications in soil nutrient stoichiometry, thereby affecting the microbial activity and nutrient availability^13^,^17^. Importantly, the shift in soil bacterial communities and the soil N fractions following afforestation and the interactions among these components may in turn provide feedback to N stock and plant productivity.

The Loess Hilly Region (LHR) of China, with severe soil erosion and low vegetation coverage^19^, plays a vital role in environmental changes including plant composition, biodiversity^20^, soil water conditions^21^, soil nutrient conditions^8^,^22^. To counteract soil erosion and nutrient loss, the Chinese government has implemented environmental protection policies since 1950^23^, including the “Grain to Green Project” (GTGP), which is a large-scale ecological rehabilitation project that has been ongoing since 1999 and has achieved initial success with more than 9.27 million ha of farmland afforested in LHR^24^. Several studies were mainly focused on in the physical and chemical properties of soil^25^,^26^ as well as biological features such as soil enzyme activity and soil microbial biomass after afforestation^26^,^27^. Thus far, little research has applied high throughput sequencing technology to assess the soil microbial diversity following afforestation, and the possibility that soil N fractions to afforestation as well as their relationship to soil bacterial communities remain unclear.

Here, we explored the effect of afforestation on the soil bacterial communities and the N fractions in the Loess Hilly Region (LHR) of China. We hypothesized that afforestation would result in changes in the soil bacteria, including bacterial abundance and diversity, these observed increase would be driven by above-ground inputs and below-ground soil parameters^28^. Coupled with increased soil N fractions following afforestation, we expected that changes in the specific bacterial species such as Rhizobiales, the branches of Alphaproteobacteria, would result in increased soil N stocks and fractions, especially for DON^16^. Therefore, the objectives of this study were as follows: 1) to study the effect afforestation on soil bacterial abundance, diversity and compositions; 2) to study the effect of afforestation on N stock and fractions; and 3) to explore the response of soil bacteria to the soil N fractions using the “best” model selection procedure.

## Results

### Changes in the TN stocks and fractions

**Figure 1:**
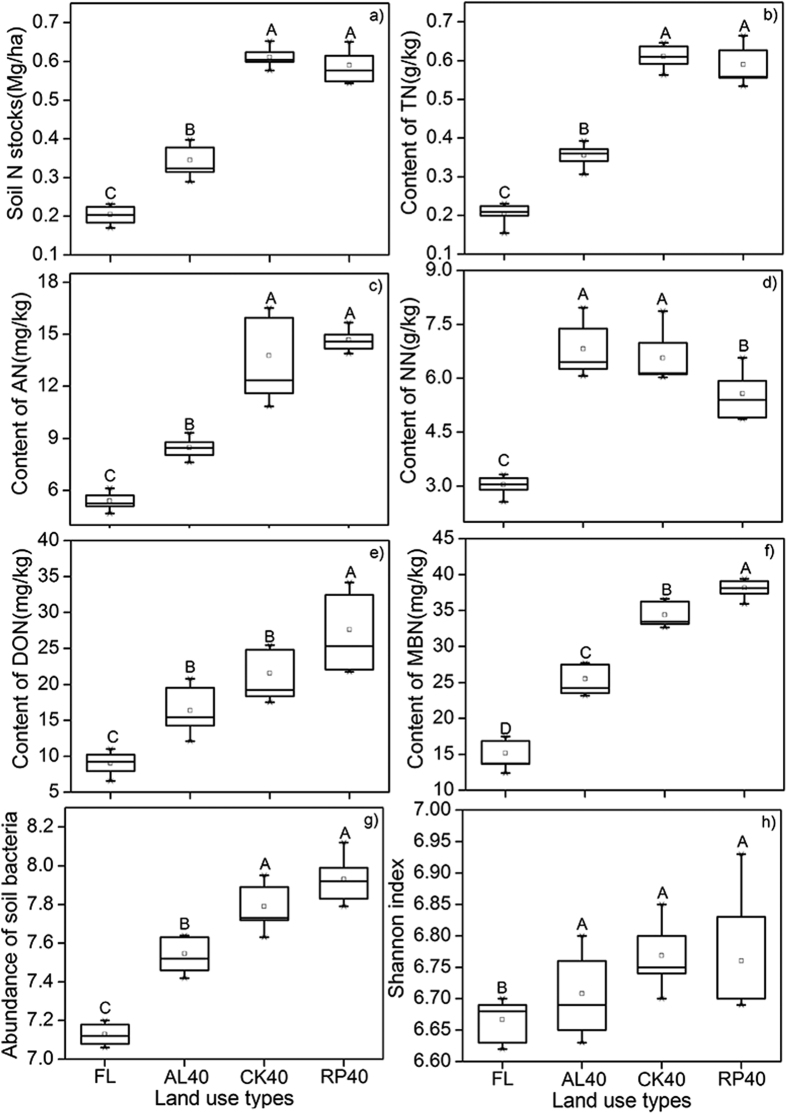
Changes of soil nitrogen (N) stocks, soil nitrogen (N) fractions. Different capital letters indicate significant differences among different land use types (P < 0.05). The error bars show standard errors.

The total nitrogen (TN) content and stocks at a depth of 0–10 cm in RP40, CK40 and AL40 were higher by 65.08–188.61% and 68.67–201.65%, than those in FL (Fig. 1a,b), respectively. Compared with FL, the ammonium nitrogen (AN), dissolved organic nitrogen (DON) and microbial biomass nitrogen (MBN) contents in RP40 were more than 83.22–204.21% higher, and similar trends were also found in CK40 and AL40 (115.87–156.33% and 57.49–124.26%, respectively) (Fig. 1c,e,f); however, the NN content was higher in AL40. The DON contents responded more sensitively than the other nitrogen fractions to afforestation (Similarity Percentages: SIMPER; average cumulative explanation of the difference in DON was 34.19%, respectively).

### Changes in soil bacterial abundance, diversity and composition

**Figure 2:**
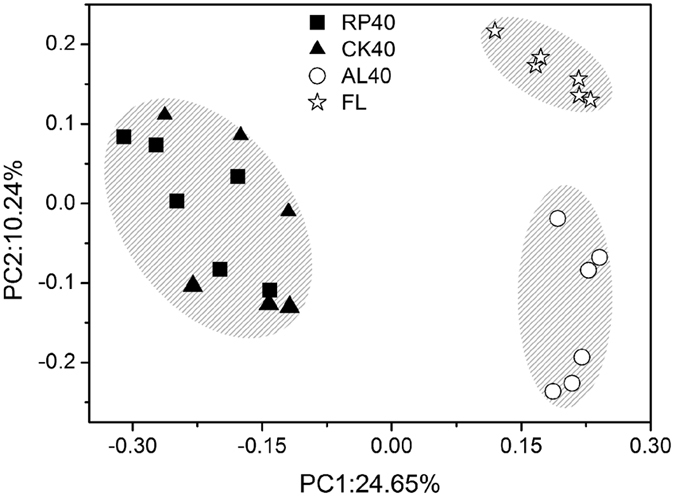
Unweighted-Unifracted PcoA of soil bacterial communities following afforestation.

The soil bacterial 16S rRNA gene copy abundance differed significantly (p < 0.05) and displayed an increasing trend from FL to RP40 after afforestation (Fig. 1g). The abundance of soil bacteria in RP40 was not significantly different from that in CK40, but greatly differed from that in AL40 and FL. Compared with the farmland, the alpha diversity (Shannon, H) in RP40, CK40 and AL40 significantly increased by 1.4%, 1.525% and 0.625%, respectively (P < 0.05) (Fig. 1h). The beta diversity of the soil bacterial communities was highly variable with respect to afforestation (Adonis: R_2_ = 0.286, P = 0.001), the soil bacterial communities between replicates in RP40 were more similar to that in CK40 than AL40 and FL, and both aggregation degrees of the sample points in AL40 were far away from that in FL (unweighted -Unifracted PCoA) (Fig. 2).

**Figure 3:**
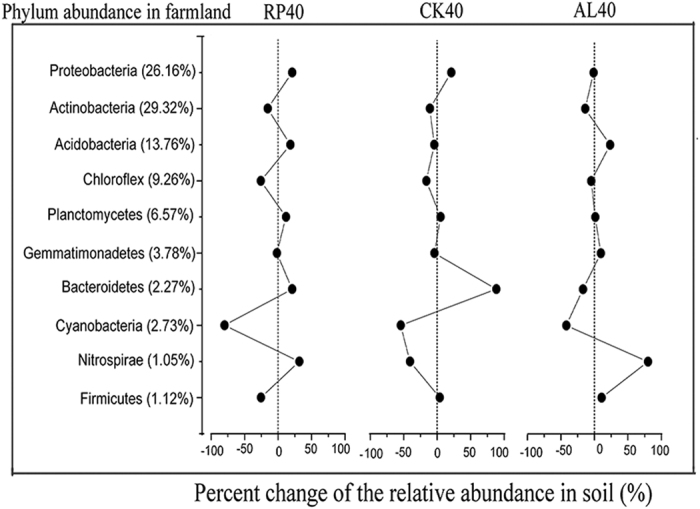
Distribution of 16S rRNA sequences across bacterial phyla communities following afforestation.

**Figure 4:**
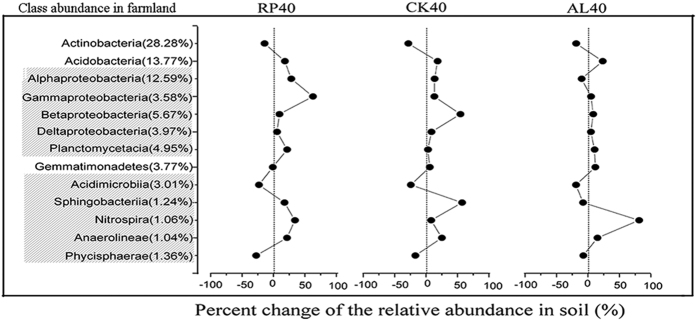
Distribution of 16S rRNA sequences across bacterial class communities following afforestation.

Changes in the relative abundance of specific taxa of soil bacteria at the phylum and class level were also observed (Figs 3 and 4 and Supplementary Tables S2 and S3). At the phylum level (average values >= 1%), *Proteobacteria, Actinobacteria* and *Acidobacteria* were the most abundant and changed intensely in response to afforestation (Fig. 3), with average contributions of 28.7%, 25.4% and 15.80%, respectively. The abundance of *Actinobacteria* in the farmland was higher than that in the forest, and the *Proteobacteria* and *Acidobacteria* in RP40, CK40 and AL40 were more abundant than in the farmland. However, the average abundance of Acidobacteria were higher in AL40 than in RP40 and CK40. Other phylum species, including *Chloroflexi* (F_3,23_ = 5.889, P = 0.005), Cyanobacteria (F_3,23_ = 18.636, P < 0.001) and *Nitrospirae* (F_3,23_ = 3.624, P = 0.031) were also affected by afforestation. Furthermore, at the class level, compared with farmland, increases in the abundance of most class groups (average values >= 1%) in response to conversion were observed except for *Acidimicrobia* and *Phycisphaerae* (Fig. 4). Practically speaking, within Proteobacteria, the *Alphaproteobacteria* were the most dominant class (Fig. 4), and the *Gammaproteobacteria*, coupled with *Alphaproteobacteria*, were extremely significantly influenced by the conversion of farmland to forest (P < 0.001). Furthermore, *Acidimicrobia* and *Nitrospirae* were significantly changed (p < 0.05).

### Relationship between soil N fractions and soil bacteria

**Figure 5:**
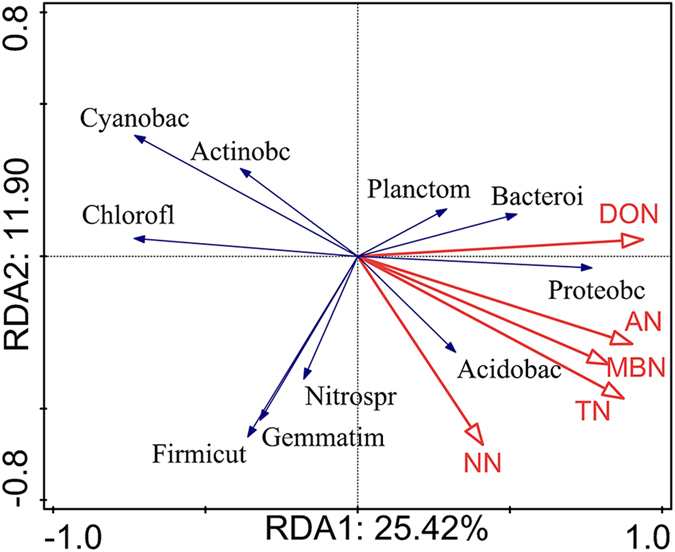
RDA analysis between soil N fractions and soil bacterial phyla following afforestation. The abbreviation were as follow: Proteobacteria (Proteobac), Actinobacteria (Actinoba), Acidobacteria (Acidobac), Chloroflexi (Chlorofl), Planctomycetes (Planctom), Gemmatimonadetes (Gemmatim), Bacteroidetes (Bacteroi), Cyanobacteria (Cyanobac), Nitrospirae (Nitrospi), Firmicutes (Firmicut), Armatimonadetes (Armatimo), Verrucomicrobia (Verrucom).

**Figure 6:**
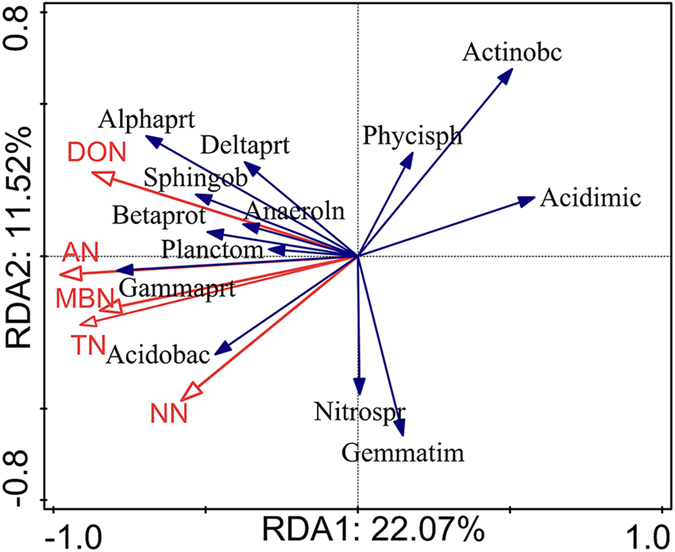
RDA analysis between soil N fractions and soil bacterial class following afforestation. The abbreviation were as follow: Actinobacteria (Actinoba), Acidobacteria (Acidobac) Alphaproteobacteria (Alphapro), Planctomycetacia (Planctom), Gammaproteobacteria (Gammapro), Betaproteobacteria (Betaprot), Deltaproteobacteria (Deltapro), Gemmatimonadetes (Gemmatim), Acidimicrobiia (Acidimic), Sphingobacteriia (Sphingob), Nitrospira (Nitrospi), Anaerolineae (Anaeroli), Phycisphaerae (Phycisph).

Significant correlations existed among the soil N fractions, bacterial abundance and alpha diversity (Shannon index, H) (P < 0.01), except for the correlation between alpha diversity and NN content (p = 0.053). Moreover, the changes in beta diversity were mainly due to changes in the relative abundances of specific bacteria taxa after afforestation, which were related to the soil N fractions (Figs 5 and 6 and Table 1). After analysis with the “best” model method, the current results showed that DON was the single best predictive factor of changes of specific species at the phylum and class level among the soil N fractions after afforestation (Table 1). Rredundancy analysis (RDA) showed that the changes in DON, MBN and AN were positively correlated with the abundance of Proteobacteria and were negatively correlated with the abundance of both Cyanobacteria and Chloroflexi (Fig. 5), whereas N fractions, except for NN, were also sensitive to the changes in the relative abundance of Alphaproteobacteria at the class level (Fig. 6).

## Discussion

The change in land use from agriculture to forestry imply that the annual cycle of cultivating and harvesting crops was replaced by a considerably longer forest cycle^23^. As a result, the biogeochemically-relevant soil physicochemical properties can change due to the process of afforestation, which in turn can influence N dynamic^6^,^9^,^29^. Consequently, Mikkelson, *et al*.^30^ reported that hydrobiogeochemical processes are important for the growth of microbes and nutrient cycling. Afforestation of former land with RP and CK and the resultant terrestrial alterations are coupled to shifts in above- and below-ground microbial communities^31^,^32^,^33^. During the process of afforestation in our study area, increased soil N stocks were evidenced by increased litter inputs relative to farmland, and changes in soil properties, such as pH and SWC (Table 2 and Supplementary Table S1). It is possible that higher litter and root biomass in afforested ecosystems than farmland may influence the decomposition of organic matter^12^,^27^, further increase the N stocks and cycles^6^,^34^; meanwhile, the difference in soil water content and pH^35^,^36^, caused by land use, could also affect the mineralization rate of organic matter and N cycling, and as result alter N stocks^9^,^37^. Therefore, soil N stocks were higher in afforested soil (RP, CK, and AL), suggesting that cumulative changes in litter (litter and root biomass) and differences in soil parameters such as pH and water content can alter soil organic matter and possibly increase soil N stocks following afforestation.

Soil N fractions can be presented as N-containing compounds (amino acids, amino sugars, and nucleic acids), which vary with litter deposition, roots, and plant compositions^12^,^16^,^18^. The present study showed that DON responded more significantly than the other N fractions following afforestation. Hence, it would suggest that DON, accounting for a large proportion of total soluble N stocks^29^, could easily be influenced by the above- and below-ground changes during afforestation. A subsequent study also evidenced that most forest soil N (96–98%) contained in dead organic matter included complex insoluble polymers such as proteins, nucleic acids, and chitin, and these polymers are mainly broken down into DON^29^,^38^. Therefore, higher litter and root biomass in afforested systems may increase the availability of more insoluble polymers from altered dead organic matter inputs, resulting in higher DON contents.

Meanwhile, changes of the above-ground inputs and below-ground soil parameters also contributed to the differentiation of the soil microbial community^28^,^39^. Our study showed that soil bacterial communities differed significantly by afforestation, which were associated with decomposing needle litter and were coupled to changes in ecological parameters such as pH and SWC (Supplementary Table S1). Notably, soil bacterial abundance (16S rRNA gene copy) and alpha diversity (Shannon, H) increased after afforestation. A possible explanation is that the nutrient resources, such as litter and root biomass, in the afforested ecosystems (RP, CK, and AL) were more abundant than those in the farmlands, and could provide more sufficient levels of nutrients for bacterial growth^11^. Compared with farmland, the amount of below-ground roots in RP and CK could liberate available nutrients associated with rhizosphere processes^27^ (i.e., forests have larger roots that farmland), thereby promoting the increase of community diversity^11^,^40^. This result was also evidenced by previous findings that plant nutrients imported to the soil in the form of litter and root exudates, and specifically selected for heterotrophic microbial communities^40^,^41^. Meanwhile, as soil bacterial diversity changed after afforestation (Figs 1 and 2), the relative taxonomic compositions of the soil bacteria differed significantly (Supplementary Tables S2 and S3 and Figure S1), suggesting that some specific clades were the primary contributors of soil microbial community structures. This phylum-level profile was similar to those in other soils and environments^42^,^43^. Regardless of afforestation, three dominant phyla, *Proteobacteria*, *Acidobacteria*, and *Actinobacteria*, were frequently discussed in previous studies^43^,^44^,^45^,^46^ and reflected the soil conditions^28^,^47^. Many soil *Proteobacteria* have been described as fast growing copiotrophs that thrive in environments with high carbon availability, whereas *Acidobacteria* and *Actinobacteria* were oligotrophic groups that preferred nutrient-poor environments^47^,^48^. In the current study, soil bacterial compositions generally changed from *Actinobacteria* (*Acidobacteria*)-dominant in farmland to *Proteobacteria*-dominant in afforested soil (Figure S2), similarly to the increased soil nutrients after afforestation^32^, indicating that the soil bacterial community transitioned from oligotrophic to copiotrophic groups. Torsvik & Øvrea^49^ also reported that these ratios were lower in oligotrophic soils and higher in copiotrophic soil with organic input. Hence, our study demonstrated that changes of soil dominant phyla (class) strongly modified soil bacterial diversity, and resultant alterations in soil may reflect the improvement of soil nutrient status after afforestation.

Soil microbial communities mediate ecological processes such as litter decomposition and mineralization^16^,^41^, causing considerable changes in soil N dynamics^41^ and ultimately resulting in an alteration to N cycling^3^. In the present study, soil bacterial diversity and dominant phyla were significantly correlated with soil N fractions, suggesting that increased soil N fractions after afforestation are responsible for the shift of soil microbial community structure. Interestingly, our study also revealed that DON was the most predictive factor structuring bacterial communities among N fractions, possibly since the accumulation of soil organic matter following afforestation may be broken down into DON through extracellular enzymes that are produced by soil microbes^12^. Farmland regenerated with plants could absorb more DON directly from the soil in the form of amino acids, through microbial mineralization^16^. Fierer, *et al*.^50^ also revealed that once N has access to DON pool, DON may be recycled and reused in microbial systems through free-living soil microbes and mineralization-immobilization of microbial biomass.

Additionally, soil microbes change the N cycling and availability by transforming N to more mobile forms^12^,^16^ and different N forms in soil are correlated with different bacterial processes that are involved in N cycling^51^. Thus, similarities in this study, increased soil N forms including DON, AN, and MBN, could suggest higher activity of nitrogen-fixing bacteria and ammonia oxidizing bacteria in forests^29^,^51^,^52^. The more significant orders under the four land use types belonged to *Rhizobiales* and *Xanthomonadales,* both of them belong to Proteobacteria phylum (Supplementary Table S4, Figure S2), which are associated with N fixation^16^. In particular, *Rhizobiales*, which are heterotrophic and nitrogen-fixing organisms^16^, were the most represented among the *Alphaproteobacteria* in afforested soil (RP, CK, 72% each) and corresponded to increased N fractions (Supplementary Table S5, Fig. 1). In contrast, changes in *Cyanobacteria* were negatively correlated with soil N fractions (DON, AN, and MBN) in the present study, which was inconsistent with previous studies^16^,^53^ showing that nitrogen-fixing *Cyanobacteria* contribute to N accumulation in terrestrial ecosystems. Competition for nutrients with large *Rhizobiales* in afforested soil (RP, CK, AL) may explain the decreased abundance of *Cyanobacteria* we observed following afforestation. However, NO_3_^−^-N did not determine the soil bacterial compositions. This result is inconsistent with a previous study where NO_3_^−^-N played an important role in driving soil bacterial compositions during the secondary succession of abandoned land in the Loess Plateau^28^; however, both findings indicated that NO_3_^−^-N could impact soil bacterial compositions in abandoned land. It is possible that forests compete with soil microbes for more NO_3_^−^-N^12^, resulting in a weak relationship between NO_3_^−^-N and soil bacterial compositions. Both NO_3_^−^-N and the relative abundance of *Nitrospira* were higher in abandoned land than other land use types, also supporting the changes of NO_3_^−^-N following afforestation. Consequently, our study demonstrated a relationship between soil N fractions and bacterial compositions, suggesting that soil N fractions changed with different soil bacterial compositions following afforestation. Although our study provides a scientific and reasonable explanation for the nitrogen fractions and the composition of soil bacteria following afforestation, there are also some uncertain factors that drove the changes in the underlying relative abundance of species, including temporal variability, with a snapshot and over time, perturbed environments, and more species colonized or that came out of dormancy. These factors are essential to discuss in a future study.

## Conclusions

Our study documented that afforestation of former farmland, apart from influencing the above-ground plant compositions, resulted in below-ground that were characterized by changes of soil properties (pH, SWC), N stocks, N fractions (TN, AN, NN, DON, MBN) and soil microbial community. Shifts in inputs with changes of plant compositions can increase the N stocks and fractions following afforestation. Similarity, soil bacterial abundance and diversity were also related with increased litter and root biomass. Changes of three dominant phyla such as *Proteobacteria, Actinobacteria,* and *Acidobacteria* could reflect the soil nutrients status after afforestation. Furthermore, N fractions were also correlated with soil bacterial phylum, especially for DON. Increases of other fractions also depended on changes of soil bacterial species. Thus, apart from the above inputs, the changes in the soil bacterial communities should be taken into account when assessing the effect of afforestation on the soil N stock and fractions on the Loess Hilly Region (LHR) of China and vice versa. In the future, more attention should be paid to plant-soil-microbe interactions from aboveground and belowground during N cycling.

## Methods

### Study area

**Figure 7:**
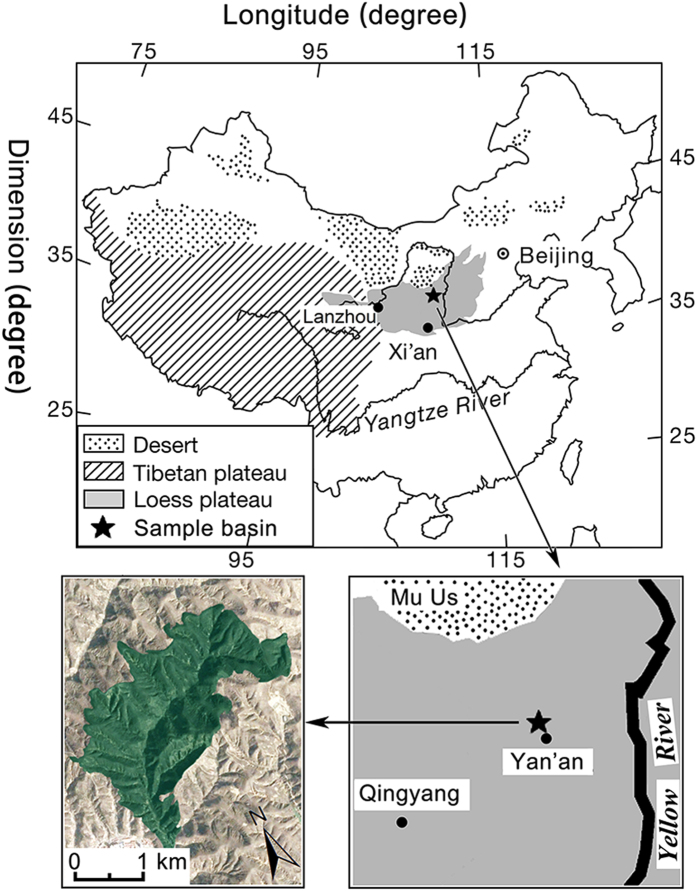
Location of the Loess Plateau and the study basin, Maps generated using ArcGIS (10.0) (http://www.esri.com/software/arcgis).

This study was conducted in the Wuliwan catchment, located in the middle area of the Loess Plateau (36°51′41.23″–36°52′50.87″N, 109°19′49.20″–109°21′46.46″E) (Fig. 7). The study site is a temperate semiarid area with average annual temperature of 8.8 °C and average annual precipitation of 510 m (mainly from July to September). Moreover, the accumulated temperatures above 0 °C and 10 °C were 3733 °C and 3283 °C, respectively. The frost-free period is 157 days, and the sunny time is 2415 h per year. The soil is mainly highly erodible Huangmian soil (Calcaric Cambisols, FAO).

The study catchment, the Wuliwan catchment, has been an experimental site of the Institute of Soil and Water Conservation, Chinese Academy of Science (CAS) since 1973. Widespread vegetation restoration has been implemented to remedy the soil degradation problem during the past decade. As shown in Fig. 7, samples were collected from farmlands mainly replanted with *Robinia pseudoacacia* L. (RP) or *Caragana Korshinskii* Kom (CK) and from abandoned land. Details of four land use types are provided in Table 2 and Supplementary Table S5, which were determined in April 2014, including litter-N (mean 19.41 g/kg), FRB (mean 1126.03 kg.hm^−2^), fine root N (mean 13.19 g/kg), pH (mean 8.39) and SWC (mean 15.28%); all the data in our study showed an increasing trend except pH.

### Sample collection

Sampling was carried out in April 2014. Based on the vegetation restoration project, three 40-year-old land types, *Robinia pseudoacacia* L. (RP40), *Caragana Korshinskii* Kom (CK40), abandoned land (AL40), and one farmland (FL) planted with millet (Setaria italica) were selected. Moreover, both *Robinia pseudoacacia* L. (RP) and *Caragana korshinskii Kom*. (CK) have been widely restored on the Loess Plateau. The abandoned land (AL40) that was afforested from farmland was not tilled for 40 years. For each land use type, we sampled three replicates for a total of 12 forested sites (three 25 m × 50 m), and in each site (25 m × 50 m), we designed two plots with sizes of 25 m × 25 m, which were also located on the same physiographic units; prior to afforestation, all the plots were essentially farmlands.

A total of 24 samples (4 land use types × 3 sites × 2 plots) were collected from the top 0–10 cm using a soil auger (diameter 5 cm) from the four land use types. Briefly, after removing the litter layer, we collected 10 samples with an “S” shape and incorporated spatial heterogeneity at the plot scale, for a total of six replicate soil samples per land use type. The samples were immediately sieved through 2-mm mesh to eliminate large rocks and roots. A part of the soil samples to be used for DNA extraction were transported from field to laboratory in ice bags and then stored in the laboratory at −80 °C. A second portion to be used to determine the total nitrogen were air-dried and stored at room temperature; another part of the soil samples to be used to determine the other N fractions were stored at 4 °C. Additionally, three soil profiles were dug randomly in each plot and were sampled in each sampling interval to determine the soil bulk density.

### Soil chemical properties

The total nitrogen (TN) (g.kg^−1^) was determined using the Kjeldahl method, and the contents of ammonium nitrogen (AN) (mg.kg^−1^) and nitrate nitrogen (NN) (mg.kg^−1^) were determined using KCl digestion, whereas the dissolved organic nitrogen (DON) was determined using potassium peroxy disulfate oxidation before the treated liquids were assessed by an Automated Chemistry Analyzer^54^, and the soil microbial nitrogen (MBN) (mg.kg^−1^) was measured using the chloroform fumigation extraction method^55^.

### Calculation of the N stocks

The N stocks represented the soil total N (STN) storage at each sampling depth. The N stocks of different sampling depths were calculated as follows:


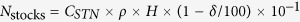


where N stocks is the density (Mg·ha^−1^) of TN, C_STN_ is the content (g·kg^−1^) of STN, ρ is the bulk density (g·cm^−3^), H is the soil horizon thickness (cm), and δ is the fraction (%) of gravels >2 mm in size in the soil. Because the soil gravel size of loess in China is mostly below 2 mm, this fraction was assumed to be 0^56^.

### Soil DNA extraction

DNA was extracted from 0.5 g of each fresh soil sample and homogenized from 10 points with E.Z.N.A soil DNA (OMEGA, USA). The concentration and quality of the DNA were measured using a spectrophotometer (NanoDrop2000, ThermoScientific, Wilmington, DE, USA). The extracted soil DNA was stored at −80 °C for PCR amplification and analysis.

### Quantitative PCR

Bacterial 16S rRNA copy numbers were estimated by primer set 515F (5′-GTGCCAGCMGCCGCGG-3′) and 907R (5′-CCGTCAATTCMTTTRAGTTT-3′)^57^. The abundance of bacterial small-subunit rRNA gene copies were quantified against standard curves generated from a 10-fold serial dilution of cloned full-length copies of either 16S rRNA gene. The reactions were carried out using an ABI Prism 7900 Sequence Detection System (Applied Biosystems, USA). Quantification was based on the fluorescence intensity of the SYBR Green dye during amplification. The 20 μl qPCR reactions contained 10 μl EvaGreen 2X qPCR MasterMix (Applied Biological Materials Inc., Richmond, Canada), a final concentration of each primer of 0.5 μM, and an environmental and standard DNA template at 1 μl per reaction to bacteria. The plates were run with the following cycle: 40 cycles of 95 °C for 15 s, 53 °C for 30 s and 72 °C for 60 s. All of the qPCR reactions were run in triplicate with each DNA template. The amplification efficiency of the qPCR was 86–97% (R^2^ > 0.992).

### PCR amplification and sequencing of 16S rRNA genes

PCR amplification of the bacterial 16S rRNA targeting the V4 region^58^ was conducted. The primers were 515F and 907R^57^. The PCR reaction system contained 0.4 μl of the two primers, 0.4 μl of FastPfu polymerase, and 10 ng 1.25 μl of template DNA. After preparation, the samples were denatured at 95 °C for 3 min, then amplified using 27 cycles of 95 °C for 30 seconds, 55 °C for 30 seconds, and 72 °C for 45 seconds, followed by an extension at 72 °C for 10 min. Each sample was amplified three times. PCR products were extracted from 2% agarose gels and purified using the AxyPrep DNA Gel Extraction Kit (Axygen Biosciences, Union City, CA, US). Finally, according to the manufacturer’s instructions, an equal amount of PCR product from each sample was combined in a single tube according to the manufacturer’s instructions and sent to Illumina’s MiSeq platform at the Major Biological Institute in Shanghai, China.

### Processing of 16S rRNA gene data

The reads were demultiplexed, quality-filtered, and processed using QIIME and based on three criteria. First, the 300-bp reads were truncated at any site with an average quality score < 20 over a 50-bp sliding window. The truncated reads that were shorter than 50 bp were then discarded. Second, exact barcode matching, two-nucleotide mismatches in primer matching, and reads containing ambiguous characters were removed. Third, only sequences that overlapped longer than 10 bp were assembled according to their overlap sequence. Reads that could not be assembled were discarded. Operational Taxonomic Units (OTUs) were clustered with a 97% similarity cutoff using UPARSE (version 7.1 http://drive5.com/uparse/), and chimeric sequences were identified and removed using UCHIME. The taxonomy of each 16S rRNA gene sequence was analyzed by RDP Classifier (http://rdp.cme.msu.edu/) against the Silva (SSU115) 16S rRNA database using a confidence threshold of 70%. Finally, the complete dataset was sent to the Sequence Read Archive (SRA) database of the National Center for Biotechnology Information (NCBI) under the accession number of SRP056716.

### Bioinformatics analysis

The calculated indices related to alpha diversity, including the Simpson index and Shannon index, which were determined by mothur (version v.1.30.1), were used to show the community diversity. Unweighted-Unifracted PcoA was performed to assess the cluster of soil bacterial communities according to land use types^44^. MEGAN was used to determine the phylogenetic analysis of the bacteria after conversion^59^. The similarities were determined to test whether there were significant differences in the microbial communities among the four land use types using “Adonis,” a vegan package in the R environment.

Pearson correlations among litter, litter-N, fine root biomass, fine root-N, soil N fractions, bacterial abundance and alpha diversity (Shannon index, H) were determined using the SPSS 22.0 software package. The linear regression analysis between the PcoA 1 and the SWC and pH were calculated using the SPSS 22.0 software package. The changes in the soil N stocks and fractions and the soil bacterial abundance and diversity among the land use types were tested with One-Way ANOVA using the R v.3.1.3 program. All p-values were considered significant if at or below the 0.05 threshold. Additionally, we used the “best” model building procedure in PRIMER v.7 to identify all possible combinations of factors that contributed to the highest proportion of soil bacterial phylum and other species; factor addition was evaluated stepwise and was based on sufficient improvement in the model’s R^60^. Redundancy analysis (RDA) was used to identify the relationship between the soil specific bacterial species and N fractions. This analysis was performed using the CANOCO 4.5 software package^61^.

## Additional Information

**How to cite this article**: Ren, C. *et al*. Responsiveness of soil nitrogen fractions and bacterial communities to afforestation in the Loess Hilly Region (LHR) of China. *Sci. Rep.* **6**, 28469; doi: 10.1038/srep28469 (2016).

## Supplementary Material

Supplementary Information

## Figures and Tables

**Table 1:** Results from “best” model selection procedure presented for each number of predictor variables of specific species at the phylum and class level (Relative abundance > 1%).

Phylum level			Class level		
Number variables	R	Predictor variables	Number variables	R	Predictor variables
Global Test	R^a^ = 0.417	P^c^ = 3.3%	Global Test	R^b^ = 0.496	P^d^ = 0.9%
1	**0.417**	**DON**	**1**	**0.448**	**DON**
2	**0.371**	**DON, MBN**	**2**	**0.496**	**DON, MBN**
2	0.337	TN, DON	2	0.377	TN, DON
2	0.319	AN, DON	2	0.376	AN, DON
2	0.196	NN, DON	3	0.470	AN, DON, MBN
3	0.354	AN, DON, MBN	3	0.385	TN, DON, MBN
3	0.300	TN, DON, MBN	3	0.339	NN, DON, MBN
3	0.271	TN, AN, DON	3	0.304	TN, AN, DON
3	0.225	NN, DON, MBN	4	0.321	TN, AN, DON, MBN
4	0.229	TN, AN, DON, MBN	4	0.308	AN, NN, DON, MBN
5	0.117	TN, AN, NN, DON, MBN	5	0.251	TN, AN, NN, DON, MBN

Number of permutations: 999 (Random sample). ^a,b^Means the Sample statistic (Average R); ^c,d^Means the significance level of sample statistic.

**Table 2:** Characteristics of four land use types.

Land use^a^	Age (y)	Location	Elevation (m)	Slope aspect (°)	Litter-N^b^ (g/kg)	FRB^c^ (kg•hm^−2^)	FRB-N^d^ (g/kg)	pH	SWC^e^ (%)
*Robinia pseudoacacia L*	40	36.87N, 109.34E	1320	NbyE45	24.03 (±0.56)	1350.75 (±20.80)	17.70 (±0.28)	8.31 (±0.01)	20.20 (±1.16)
*Caragana Korshinskii Kom*	40	36.87N, 109.35E	1318	NbyE10	25.49 (±1.07)	1439.49 (±17.70)	14.54 (±0.34)	8.33 (±0.01)	16.21 (±0.85)
Abandoned land	40	36.87N, 109.35E	1308	NbyE30	8.72 (±1.37)	587.86 (±17.29)	7.32 (±0.14)	8.45 (±0.01)	14.62 (±0.35)
Farmland	–	36.87N, 109.35E	1273	SbyW50	No data			8.48 (±0.01)	10.10 (±0.94)

^a^Means the conversion of farmland to different land use types, including *Robinia pseudoacacia* L (RP40a), *Caragana korshinskii* Kom (CK40a) and abandoned land (AL40a), Farmland, the original land use before vegetation restoration, served as the control in our study. ^b,c,d,e^Represents the changes of litter nitrogen, fine root biomass, fine root nitrogen and soil water content after afforestation, which were determined in April 2014.
