# Supplementary material for: Responsiveness of soil nitrogen fractions and bacterial communities to afforestation in the Loess Hilly Region (LHR) of China
Source: Sci Rep. 2016 Jun 23;6:28469. doi: 10.1038/srep28469 (PMC4917850; doi:10.1038/srep28469)

Supplementary Information

Title: Responsiveness of soil nitrogen fractions and bacterial communities to afforestation in the Loess Hilly Region (LHR) of China

Authors:

Chengjie Ren[1,2],Pingsheng Sun[1,2],Di Kang[2,3],Fazhu Zhao[1,2], Yongzhong Feng[1,2] & Guangxin Ren[1,2],Xinhui Han[1,2], Gaihe Yang[1,2]

Affiliations:

[1]College of Agronomy, Northwest A&F University, Yangling, 712100 Shaanxi, China

[2]The Research Center of Recycle Agricultural Engineering and Technology of Shaanxi Province, Yangling 712100 Shaanxi, China

[3]College of Forestry, Northwest A&F University, Yangling, 712100 Shaanxi, China

Author contributions

X.H.H. and G.H.Y. conceived and designed the experiments. C.J.R, P.S.S, and F.Z.Z. Carried out the experiments. D.K plot the figures. C.J.R wrote the paper. Professor G.X.R And Y.Z.F worked on text and language correction. All authors reviewed the manuscript.

Competing financial interests.

No conflict of interest exits in the submission of this manuscript, and manuscript is approved by all authors for publication. All the authors have approved the manuscript that is enclosed.

*Corresponding author:

Xinhui Han, Gaihe Yang

College of Agronomy, Northwest A&F University, Yangling, 712100 Shaanxi, China

The Research Center of Recycle Agricultural Engineering and Technology of Shaanxi Province, Yangling 712100 Shaanxi, China

E-mail: hanxinhui@nwsuaf.edu.cn; ygh@nwsuaf.edu.cn

Tel: 13709129773

For field sampling, environmental parameter measurements and MiSeq sequencing, please contact:

Rencj1991@nwsuaf.edu.cn

Supplementary Table-S1. Pearson linear correlation coefficients among the characteristics at each site. N stocks: nitrogen stocks; TN: total nitrogen; AN: ammonium nitrogen; NN: nitrate nitrogen; DON: dissolved organic nitrogen; MBN: microbial biomass nitrogen; litter-N: litter nitrogen; RB: root biomass; RB-N: root biomass nitrogen; SWC: soil water content; PcoA 1 means the ordination of Principal coordinates analysis (PcoA). In our study, we use PcoA1 as the beta diversity based on Bray–Curtis on OUT; ASB: abundance of soil total bacteria.

	N stocks	TN	AN	NN	DON	MBN	Litter-N	RB	RB-N	pH	SWC
N stocks											
TN	.995**										
AN	.922**	.927**									
NN	.612**	.619**	.551**								
DON	.806**	.805**	.877**	.531**							
MBN	.937**	.934**	.918**	.618**	.869**						
Litter-N	.944**	.953**	.907**	-0.321	.628**	.867**					
FRB	.959**	.964**	.865**	-0.369	.593**	.879**	.972**				
FRB-N	.877**	.877**	.883**	-.561*	.740**	.966**	.904**	.909**			
pH	-.924**	-.925**	-.911**	-.443*	-.828**	-.910**	-.932**	-.920**	-.925**		
SWC	.779**	.775**	.839**	.495*	.877**	.818**	.547*	.503*	.702**	-.769**	
Shannon	.571**	.562**	.541**	0.399	.650**	.536**	0.292	0.4	0.311	-.500*	.513*
PcoA 1	-0.833**	-0.885**	-0.882**	-0.293	-0.787**	-0.854**	-0.922**	-0.951**	-0.952**	0.902**	-0.689**
ASB [a]	.893**	.891**	.906**	.622**	.851**	.935**	.773**	.766**	.833**	-.858**	.851**

Supplementary Table-S2. Relative abundances (average values >= 1% and standard deviations) of dominant bacterial Phylum groups according to the RDP Classifier in soils of studied alder stands. And effect of land use on relative abundance evaluated by generalized linear model (GLM). ***P<0.001, ** P <0.01, * P <0.05; Capital letters indicate significant difference among different land use types, the same for supplementary table-S2, 3, 4

**Phylum groups**	**Relative abundances (%)**				**GLM**	
	**RP40(n=6)**	**CK40(n=6)**	**AL40 (n=6)**	**FL (n=6)**	**df (3, 23)**	**Sig**
Proteobacteria	31.60±2.08 A	31.13±3.05 A	25.77±1.33 B	26.16±3.37 B	8.735	0.001**
Actinobacteria	24.74±5.05 AB	22.45±2.83 B	25.23±4.90 AB	29.32±5.14 A	2.348	0.103
Acidobacteria	16.27±1.91 A	16.21±2.24 A	16.97±0.35 A	13.76±1.50 B	4.362	0.016*
Chloroflexi	6.86±0.59 B	7.37±1.40 B	8.80±0.87 A	9.26±1.52 A	5.889	0.005**
Planctomycetes	7.35±1.70 A	6.98±0.64 A	6.65±1.53 A	6.57±1.58 A	0.373	0.774
Gemmatimonadetes	3.71±0.98 A	3.96±0.53 A	4.14±0.34 A	3.78±0.70 A	0.455	0.717
Bacteroidetes	2.74±1.08 AB	3.54±1.76 A	1.88±0.31 B	2.27±0.99 AB	2.308	0.107
Cyanobacteria	0.55±0.72 C	0.72±0.62 C	1.58±0.45 B	2.73±0.44 A	18.636	0.000**
Nitrospirae	1.38±0.33 AB	1.12±0.51 B	1.89±0.73 A	1.05±0.16 B	3.624	0.031*
Firmicutes	0.83±0.29 B	1.29±0.30 A	1.24±0.39 A	1.12±0.23 AB	2.854	0.063

Supplementary Table-S3. Relative abundances (average values >=1% and standard deviations) of dominant bacterial Class groups according to the RDP Classifier in soils of studied alder stands. And effect of land use on relative abundance evaluated by generalized linear model (GLM).

Class groups	Relative abundances (%)				GLM	
	RP40(n=6)	CK40(n=6)	AL40 (n=6)	FL (n=6)	df (3, 23)	Sig
Alphaproteobacteria	16.13±1.63 A	14.27±0.87 B	11.31±0.61 B	12.59±0.63 B	23.941	0.000***
Gammaproteobacteria	5.82±0.60 A	5.54±0.36 B	3.88±1.01 C	3.58±1.13 C	10.406	0.000***
Betaproteobacteria	6.20±0.65 A	6.15±0.96 A	5.92±0.52 A	5.67±1.00 A	0.42	0.741
Deltaproteobacteria	4.19±0.39 A	4.09±0.79 A	4.38±0.18 A	3.97±0.12 A	0.783	0.518
Planctomycetacia	6.01±1.36 A	5.59±0.32 A	5.18±1.40 A	4.95±1.17 A	0.993	0.416
Acidimicrobiia	2.31±0.39 B	2.28±0.28 B	2.44±0.34 B	3.01±0.67 A	3.402	0.039*
Sphingobacteriia	1.45±0.55 AB	1.95±0.74 A	1.14±0.24 B	1.24±0.53 B	2.626	0.079
Nitrospira	1.42±0.37 AB	1.15±0.53 B	1.93±0.72 A	1.06±0.16 B	3.808	0.026*
Anaerolineae	1.26±0.41 A	1.30±0.44 A	1.20±0.28 A	1.04±0.38 A	0.529	0.668
Phycisphaerae	0.98±0.35 A	1.13±0.45 A	1.25±0.49 A	1.36±0.41 A	0.843	0.486

Supplementary Table-S4. Relative abundances (average values >= 1% and standard deviations) of dominant bacterial Order groups according to the RDP Classifier in soils of studied alder stands. And effect of land use on relative abundance evaluated by generalized linear model (GLM).

**Order groups**	**Relative abundances (%)**				**GLM**	
	**RP40(n=6)**	**CK40(n=6)**	**AL40 (n=6)**	**FL (n=6)**	**df (3, 23)**	**Sig**
Rhizobiales	11.576±1.133 A	10.454±0.908 B	7.627±0.363 C	7.476±0.383 C	42.381	<0.001***
Solirubrobacterales	6.536±1.565 A	5.728±0.661 A	6.496±1.464 A	6.593±1.473 A	0.557	0.65
Planctomycetales	6.011±1.357 A	5.592±0.321 A	5.182±1.403 A	4.949±1.171 A	0.994	0.416
Xanthomonadales	4.370±0.608 A	4.027±0.642 A	3.000±0.779 B	2.891±1.070 B	5.158	0.008**
Gemmatimonadales	3.247±0.923 A	3.451±0.649 A	3.526±0.380 A	3.049±0.670A	0.594	0.626
Gaiellales	3.149±0.925 A	3.012±1.200 A	3.857±1.098 A	3.422±0.946 A	0.756	0.532
Rhodospirillales	2.899±0.313 A	2.138±0.728 B	2.793±0.196 A	3.228±0.524 A	5.325	0.007**
Propionibacteriales	2.574±0.666 B	2.346±0.292 B	2.109±0.420 B	3.177±0.469A	5.447	0.007**
Myxococcales	2.329±0.440 A	2.260±0.667A	2.198±0.387 A	2.777±0.288 A	1.911	0.16
Nitrosomonadales	2.316±0.643 B	2.674±0.326 AB	3.246±0.580 A	2.411±0.415 B	4.07	0.021*
Acidimicrobiales	2.306±0.391 B	2.275±0.279 B	2.287±0.478 B	3.008±0.675 A	3.391	0.038*
Burkholderiales	2.306±0.391 B	2.275±0.279 B	2.287±0.478 B	3.008±0.675 A	3.391	0.038*
Micrococcales	1.760±0.439 B	1.829±0.206 B	1.427±0.371 B	3.098±0.476 A	21.572	<0.001**
Pseudonocardiales	1.629±0.448 A	1.184±0.373 A	1.489±0.553 A	1.159±0.125 A	1.937	0.156
Frankiales	1.547±0.514 BC	1.120±0.238 C	1.803±0.553 B	3.531±0.370 A	35.229	<0.001**
Sphingobacteriales	1.455±0.544 AB	1.948±0.744 A	1.142±0.237 B	1.240±0.528 B	2.613	0.08
Micromonosporales	*1.304±0.383 A*	*1.315±0.384 A*	*1.396±0.615 A*	*1.181±0.213 A*	*0.263*	*0.851*

Supplementary Table-S5. Plants information and main species in each land use type. Grass species with important value>1%. Important value is the average of relative coverage, relative frequency, and relative density.

Plant	Species(Woody/Shrub)	Quantity	Grass	Important value
	Robinia pseudoacacia L.	92	Ulmus pumila L	10.09
	Ulmus pumila L.	8	Pennisetum centrasiaticum	8.66
	Pyrus betulifolia	28	Poa sphondylodes	8.22
	Armeniaca sibirica	1	Draba nemorosa	6.73
	Periploca sepium	65	Tripolium vulgare	6.47
	Rubus parvifolius L	12	Melica scabrosa	5.87
	Prinsepia uniflora	5	Bidens pilosa	5.81
	Rosa multiflora	4	Thlaspi arvense L	4.97
Robinia pseudoacacia L. (RP)			Roegneria kamoji	4.55
			Salsola collina	3.83
			Artemisia mongolica	3.81
			Cleistogenes hancei	2.99
			Heteropappus hispidus	2.33
			Stipa bungeana	2.11
			Viola dissecta	1.95
			Dendranthema chanetii	1.85
			Scirpus wallichii	1.79
			Cynanchum thesioides	1.40
			Cleistogenes	1.30
			Rehmannia glutinosa	1.14
			Ixeris polycephala	1.11
			Leonurus artemisia	1.04
			Heteropappus altaicus	1.01
Caragana korshinskii Kom.(CK)	Caragana korshinskii Kom	552	Tripolium vulgare	14.91
			Heteropappus altaicus	12.33
			Roegneria kamoji	9.57
			Dendranthema indicum	8.90
			Rubia cordifolia	7.28
			Melica scabrosa	5.17
			Viola dissecta	5.04
			Potentilla tanacetifolia	4.34
			Thlaspi arvense L	4.33
			Artemisia mongolica	4.27
			Artemisia capillaris	3.65
			Ixeris polycephala	3.04
			Cynanchum thesioides	2.20
			Caragana korshinskii	2.18
			Medicago	2.06
			Elymus dahuricus	2.06
			Bidens pilosa	1.99

			Bupleurum chinense	1.83
			Artemisia leucophylla	1.68
			Poa sphondylodes	1.59
			Glycyrrhiza uralensis	1.57
	None	None	Bidens pilosa	8.22
			Roegneria kamoji	7.64
			Lespedeza davurica	7.47
			Tripolium vulgare	7.26
			Thermopsis lupinoides	6.93
			Artemisia scoparia	6.46
			Bothriochloa ischaemum	6.40
			Phragmites australis	6.20
			Glycyrrhiza uralensis	4.19
			Artemisia capillaris	3.83
			Astragalus melilotoides	3.23
			Poa pratensis	2.55
Abandoned land			Dracocephalum moldavica	2.39
			Poa sphondylodes	1.94
			Vicia bungei	1.84
			Stipa bungeana	1.73
			Polygala tenuifolia	1.59
			Dendranthema indicum	1.56
			Sophora japonica	1.52
			Setaria viridis	1.32
			Heteropappus altaicus	1.24
			Potentilla tanacetifolia	1.03
			Ulmus pumila	1.01

Supplementary Figure-1. The phylogenetic analysis of bacteria after afforestation (MEGAN software was used). ***P<0.001, ** P <0.01, * P <0.05

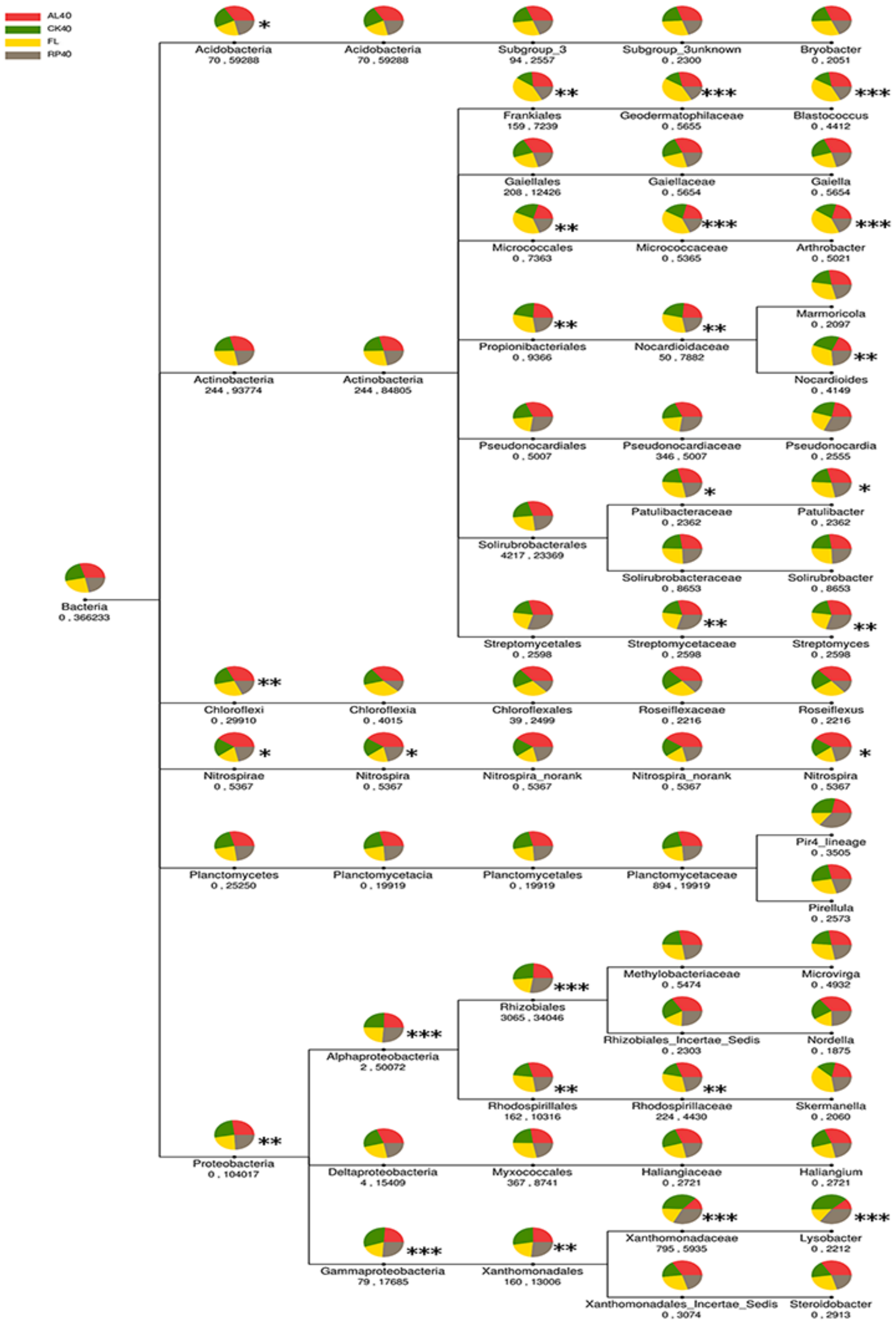

Supplementary Figure-2. Changes of both Proteobacteria-to-Acidobacteria ratio and Proteobacteria-to- Actinobacteria ratio (means±SD) among land uses

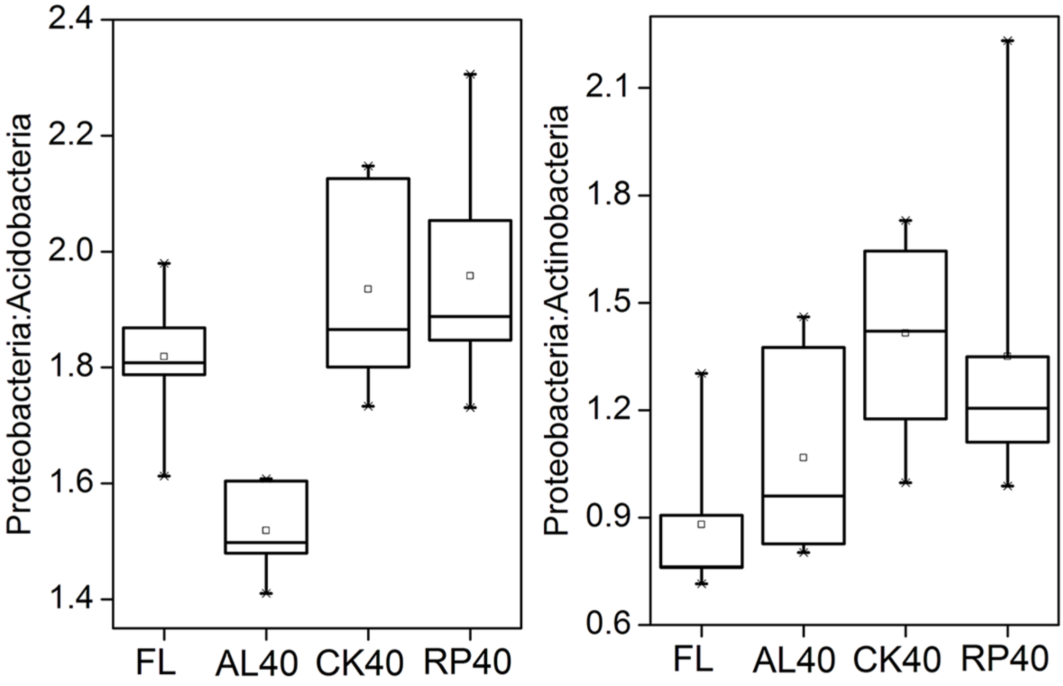

Supplement: Supplementary Information [file srep28469-s1.pdf]
